# A Hypothalamic Leptin-Glutamate Interaction in the Regulation of Sympathetic Nerve Activity

**DOI:** 10.1155/2017/2361675

**Published:** 2017-08-03

**Authors:** Hong Zheng, Xuefei Liu, Yulong Li, Kaushik P. Patel

**Affiliations:** ^1^Department of Cellular and Integrative Physiology, University of Nebraska Medical Center, Omaha, NE 68198-5850, USA; ^2^Department of Emergency Medicine, University of Nebraska Medical Center, Omaha, NE 68198-5850, USA

## Abstract

Accumulated evidence indicates that obesity-induced type 2 diabetes (T2D) is associated with enhanced sympathetic activation. The present study was conducted to investigate the role for leptin-glutamate signaling within the hypothalamus in regulating sympathetic nerve activity. In anesthetized rats, microinjections of leptin (5 ng ~ 100 ng) into the arcuate nucleus (ARCN) and paraventricular nucleus (PVN) induced increases in renal sympathetic nerve activity (RSNA), blood pressure (BP), and heart rate (HR). Prior microinjections of NMDA receptor antagonist AP5 (16 pmol) into the ARCN or PVN reduced leptin-induced increases in RSNA, BP, and HR in both ARCN and PVN. Knockdown of a leptin receptor with siRNA inhibited NMDA-induced increases in RSNA, BP, and HR in the ARCN but not in the PVN. Confocal calcium imaging in the neuronal NG108 and astrocytic C6 cells demonstrated that preincubation with leptin induced an increase in intracellular calcium green fluorescence when the cells were challenged with glutamate. In high-fat diet and low-dose streptozotocin-induced T2D rats, we found that leptin receptor and NMDA NR_1_ receptor expressions in the ARCN and PVN were significantly increased. In conclusion, these studies provide evidence that within the hypothalamic nuclei, leptin-glutamate signaling regulates the sympathetic activation. This may contribute to the sympathoexcitation commonly observed in obesity-related T2D.

## 1. Introduction

Overweight and obesity are a growing “worldwide epidemic problem.” The prevalence of type 2 diabetes (T2D) has significantly increased with the prevalence of obesity. Obesity accompanying T2D is known to be closely linked with insulin resistance and elevated sympathetic nervous system activity [1, 2]. Increased sympathetic nerve activity contributes to the onset and maintenance of cardiovascular complications such as hypertension and heart failure in T2D [3]. Various types of autonomic abnormalities in relation to the cardiovascular system have been observed in diabetic patients as well as in animal models of diabetes [4, 5]. The myriad of mechanisms linking diabetes with sympathetic overactivation is complex, multifaceted, and not clearly understood. The central nervous system plays a very important role in regulating sympathetic activation and contributing to the altered neurohumoral drive during diabetes [6–9]. The activation of the sympathetic nervous system through the central action of the adipokine leptin has been suggested as a possible major mechanism that contributes to the development of hypertension and heart failure leading to cardiovascular morbidity and mortality in T2D [8, 9].

The factors that cause the elevation in sympathetic drive are the critical keys to understanding the etiology of obesity-related diabetes. Among them, leptin is an adipocyte-derived hormone that promotes weight loss by reducing appetite and by increasing energy expenditure through sympathetic stimulation of thermogenic tissue [10]. Leptin is a 16 kDa protein released by fat cells into the blood, is able to cross the blood brain barrier to interact with its receptors in various hypothalamic nuclei to affect feeding and thermogenesis, and also induces sympathetic activation to kidneys, hindlimb vasculature, and the adrenal glands [3]. Central administration of leptin has been shown to increase renal sympathetic nerve activity (RSNA), mean arterial pressure (MAP), and heart rate (HR) in conscious rabbits [11]. Elevated levels of circulating leptin associated with obesity may contribute to the development of enhanced sympathetic outflow in T2D.

The leptin receptor is expressed in several hypothalamic nuclei including the arcuate nucleus (ARCN), paraventricular nucleus (PVN), and ventromedial hypothalamus [12]. Several neurotransmitters and neuropeptides, such as glutamate, GABA, and neuropeptide Y, have emerged as principal mediators of leptin-induced action within the hypothalamus [12]. These neurotransmitters and neuropeptides exert varying effects by different pathways. However, the specific hypothalamic pathways that can mediate the effects of leptin have not been fully elucidated. As a major excitatory neurotransmitter, glutamate has been found to modulate sympathetic nerve activity in several brain areas, including the ARCN and the PVN [13, 14]. In diabetic rats, glutamatergic tone is increased in the PVN via an upregulation of the N-methyl-D-aspartate (NMDA) type 1 (NR_1_) receptor [15]. In the hippocampus, leptin has been shown to facilitate NMDA receptor function and modulate synaptic plasticity [16]. Despite this evidence, the precise central mechanisms by which leptin-glutamate signaling contributes to altered neurohumoral drive during T2D remain unknown. The present study was conducted to investigate the role for leptin-glutamate signaling within the hypothalamus in regulating sympathetic nerve activity under normal conditions and in T2D rats.

## 2. Materials and Methods

### 2.1. Animals

Normal rats: Male Sprague-Dawley rats weighing between 325 and 350 g (age 10-11 weeks) were obtained from SASCO Breeding Laboratories (Omaha, NE). This study was approved by the Institutional Animal Care and Use Committee of the University of Nebraska and was carried out under the guidelines of the American Physiological Society and the National Institutes of Health Guide for the Care and Use of Laboratory Animals.

Type II diabetic rats: Male Sprague-Dawley rats (150–180 g, age 6-7 weeks, SASCO) were maintained in a vivarium with a 12 h light/12 h dark cycle and placed on a high-fat diet (HFD, 42% of calories are from fat, Harlan). After 4 weeks, the rats were injected with low-dose streptozotocin (STZ, 30 mg/kg in citric acid, i.p.) to induce partial insulin deficiency. The rats were then fed with HFD for additional 8 weeks [17]. The “normal diet-” fed rats with vehicle injection were used as nondiabetic controls. Postprandial plasma glucose, body weight, and food consumption were monitored weekly. Glucose levels were measured from tail bleeds with a glucometer weekly. The experiments were performed after 12 weeks of HFD-STZ induction.

### 2.2. General Surgery for Recording of Renal Sympathetic Nerve Activity and Arterial Pressure

Rats were anesthetized with *α*-chloralose (140 mg/kg, i.p.) and urethane (0.75–1.5 g/kg, i.p.). The femoral artery was cannulated for monitoring mean arterial pressure (MAP) and heart rate (HR). The femoral vein was cannulated for administration of supplemental anesthesia and 0.9% saline. The left renal nerve was isolated, and the electrical signal was recorded with the PowerLab (ADInstruments, Colorado Spring, CO) as described before [18, 19]. Basal RSNA was recorded at the beginning of the experiment. Background noise was determined by nerve activity recorded at the end of the experiment after the rat was euthanized. The RSNA was calculated by subtracting the background noise from the recorded value. The changes in integrated RSNA were expressed as a percentage from the basal value. The changes in MAP and HR were expressed as the absolute difference between the basal value and the value after injection of a drug.

### 2.3. Microinjections into the ARCN and the PVN

Rats were anesthetized with *α*-chloralose (140 mg/kg, i.p.) and urethane (0.75–1.5 g/kg, i.p.). Rats were placed in a stereotaxic apparatus. An incision was made on the midline of the scalp to expose the bregma. The coordinates of the right ARCN with reference to the bregma were calculated as being 2.3 mm posterior, 0.5 mm lateral, and 9.6–9.9 mm ventral to the dura [20, 21]. The coordinates of the right side of the PVN with reference to the bregma were calculated as being 1.5 mm posterior, 0.4 mm lateral, and 7.8 mm ventral to the dura [18, 19]. 30 minutes after the surgery, a needle (0.2 mm OD) that was connected to a microsyringe (0.5 *μ*L) was lowered into the ARCN or PVN. At the end of the experiment, monastral blue dye (2% Chicago blue, 30 nL) was injected into the brain for histological verification.

**Figure 1:**
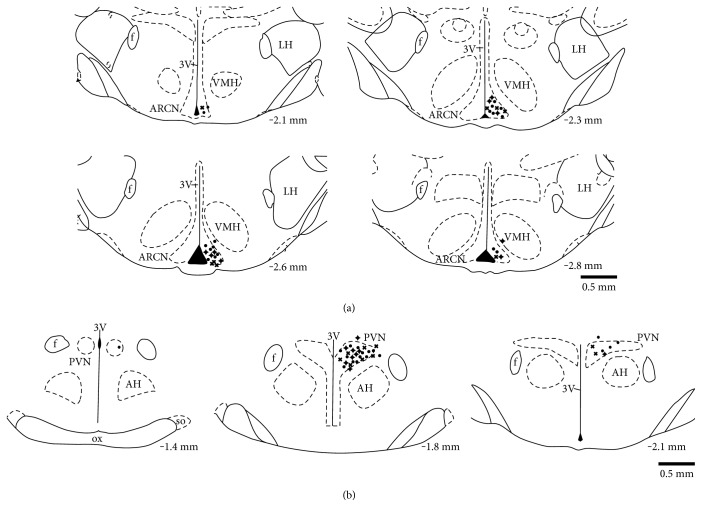
(a) Schematic representations of injection sites in serial sections from the rostral (−2.1) to the caudal (−2.8) extent of the region of the ARCN. (b) Schematic representations of injection sites in serial sections from the rostral (−1.4) to the caudal (−2.1) extent of the region of the PVN. The distance, in millimeters, posterior to the bregma is shown for each section according to Paxinos and Watson. “•” represents the center of microinjection sites from normal rats. “+” represents the center of microinjection sites from control rats. “Χ” represents the center of microinjection sites from T2D rats. LH: lateral hypothalamus; VMH: ventromedial hypothalamus; AH: anterior hypothalamus; f: fornix; 3V: third ventricle; OX: optic tract.

The brain was carefully removed and fixed in 4% formaldehyde. The brain was then frozen, and serial transverse sections (30 *μ*m) were cut using a cryostat. The sections were thaw-mounted on slides. The sections were stained using 1% aqueous neutral red. Presence of blue dye within the ARCN or PVN was determined using a light microscope. The results of these injections are shown in Figure 1.

### 2.4. Knockdown of the Leptin Receptor with siRNA

In a separated group, rats were anesthetized with ketamine (48 mg/kg, i.p.) and xylazine (12 mg/kg, i.p.). siRNA-targeting leptin receptors were purchased from Santa Cruz Biotechnology (Santa Cruz, CA). Transfection was performed using Lipofectamine 2000 (Invitrogen, Carlsbad, CA) and microinjection into the ARCN or PVN. siRNA for leptin receptors (50 nL) was delivered into the ARCN or PVN by bilateral microinjections. The coordinates of the ARCN and the PVN were described above. The skin was sutured after injection, and the rats were returned to their cages for the next 48 hours. The rats were given analgesic Buprenex injection after surgery to prevent pain.

### 2.5. Experimental Protocols

#### 2.5.1. Experiment 1

In normal rats, leptin (RD Systems, Minneapolis, MN) was microinjected (5, 25, and 100 ng in 50 nL) into the ARCN or PVN (*n* = 8 rats/group). The responses of RSNA, MAP, and HR over the following 30 minutes were recorded.

#### 2.5.2. Experiment 2

In normal rats, NMDA receptor antagonist AP5 (16 pmol in 50 nL) was microinjected into the ARCN or PVN, 10 minutes prior to microinjection of leptin (100 ng in 50 nL; *n* = 8 rats/group). The responses of RSNA, MAP, and HR over the next 30 minutes were recorded.

#### 2.5.3. Experiment 3

In a separate group of control and T2D rats, leptin receptor siRNA was microinjected into the ARCN or PVN. 48 hours after microinjection of leptin receptor siRNA, NMDA (100 pmol in 50 nL) was microinjected into the ARCN or PVN (*n* = 8 rats/group). The responses of RSNA, MAP, and HR were recorded. Negative controls (scrambled siRNA) were used in order to verify the nonspecific effects of siRNA.

### 2.6. Confocal Ca^2+^ Imaging

Cultured neuronal NG108 cells and astrocytic C6 cells (ATCC, Manassas, VA) were preincubated with leptin (5 *μ*M) for 24 hours at 37°C in a Petri dish containing laminin-coated glass cover slips. After incubation, cells were then loaded with Fluo-3 (5 *μ*M) for 30 minutes at 37°C. At the end of the incubation, cells were washed with DME medium to remove extracellular Fluo-3 and placed in a chamber on the stage of a laser confocal microscope (Zeiss Confocal LSM 510 META). The confocal calcium image with green fluorescence was taken when the neurons were challenged with glutamate (1 *μ*M). Fluo-3 was excited by light at 488 nm, and fluorescence was measured at wavelengths of >515 nm, using a 100x objective. Raw data were imported into Excel file for analysis.

### 2.7. Micropunch of the PVN for Western Blot Measurements

In a separate group of control (*n* = 6) and T2D (*n* = 6) rats, the rats were sacrificed with pentobarbital (150 mg/kg, i.p.). Then, brains were removed and frozen on dry ice. Frozen serial coronal sections (100 *μ*m/section) of the ARCN (15 sections) and PVN (6 sections) were cut with a cryostat according to a stereotaxic atlas and bilaterally punched with an 18-gauge needle using the Palkovits and Brownstein technique [22]. The punches for each brain were combined and placed in 100 *μ*L of protein extraction buffer (10 mM Tris, 1 mM ethylenediaminetetraacetic acid, 1% sodium dodecyl sulfate, 0.1% Triton X-100, and 1 mM phenylmethylsulfonyl fluoride) to extract the protein.

### 2.8. Western Blot Measurement of the Leptin Receptor and NMDA NR_1_ Receptor Protein

The total protein concentrations in the punched ARCN and PVN samples were measured with a bicinchoninic acid assay kit (Pierce, Rockford, IL). Samples were adjusted to contain the same concentration of total protein. The protein samples with 2X 4% SDS sample buffer were loaded onto a SDS-PAGE gel, subjected to electrophoresis, and then transferred to a polyvinylidene difluoride membrane (Millipore, MA). The membrane was probed with primary antibody [rabbit anti-leptin receptor (1 : 500, Abcam, Cambridge, MA), rabbit anti-NR_1_ receptor (1 : 500, Santa Cruz Biotechnology, Santa Cruz, CA), or rabbit anti-glyceraldehyde 3-phosphate dehydrogenase (GAPDH; 1 : 2000, Santa Cruz Biotechnology)] overnight and then probed with secondary antibody (peroxidase-conjugated anti-rabbit IgG, 1 : 5000, Pierce). An enhanced chemiluminescence substrate (Pierce) was applied to the membrane, followed by an exposure within an UVP system (UVP BioImaging, Upland, CA) for visualization. Kodak 1D software (Kodak, NY) was used to quantify the signal. The expression of protein was calculated as the ratio of intensity of the leptin receptor and NR_1_ receptor, respectively, relative to the intensity of GAPDH band.

### 2.9. Leptin Receptor and NMDA NR_1_ Receptor Immunohistochemistry

The rats were anesthetized with pentobarbital (65 mg/kg) and transcardially perfused with heparinized saline followed by 4% paraformaldehyde in 0.1 M sodium phosphate buffer. The brain was removed and postfixed in 4% paraformaldehyde solution and then placed in 30% sucrose. 30 *μ*m brain sections were cut with a cryostat and preserved in the cryoprotectant.

Two groups of sections (control and T2D, *n* = 4/group) were incubated with 10% normal donkey serum for 1 hour and then incubated with primary antibody against the leptin receptor (anti-goat, 1 : 200, Santa Cruz Biotechnology) or NR_1_ receptor (anti-goat, 1 : 200, Santa Cruz Biotechnology) with the neuronal marker microtubule-associated protein 2 (MAP2, anti-mouse, 1 : 200, Abcam) or glial marker glial fibrillary acidic protein (GFAP, anti-mouse, 1 : 200, BD Pharmingen, San Jose, CA) overnight at 4°C. After washing, the sections were incubated with Cy3-conjugated donkey anti-goat secondary antibody (1 : 200) and Cy2-conjugated donkey anti-mouse secondary antibody (1 : 400, Jackson ImmunoResearch, West Grove, PA) for 2 hours at room temperature. After washing and drying, the sections were cover-slipped with fluoromount-G (SouthernBiotech, Birmingham, AL). Distribution of the leptin receptor or NR_1_ receptor with MAP2 or GFAP immunofluorescence, respectively, within the ARCN and PVN was viewed using an Olympus fluorescence microscope equipped with a digital camera (QImaging, Canada).

### 2.10. Statistical Analysis

Data are presented as means ± SE. The data were subjected to one-way ANOVA followed by comparison for individual group differences with the Newman-Keuls test. Statistical significance was indicated by a value of *P* < 0.05.

## 3. Results

### 3.1. General Data

T2D was induced by a combination of both HFD and injection of low-dose STZ.  Table 1 illustrates the general characteristics of control and T2D rats used in these experiments. After 12–14 weeks of the treatments (HFD and STZ injection), the body weight and weight of the retroperitoneal fat pad were significantly higher in T2D rats. The T2D rats also show decreased brown adipose tissue. The plasma glucose level and plasma leptin were significantly higher in T2D rats than in control rats. The insulin sensitivity index was significantly decreased in the T2D rats compared with control rats. These data indicated that HFD and low-dose STZ induced hyperglycemia, hyperleptinemia, hyperlipidemia, and insulin resistance in T2D rats mimicking T2D in humans.

The basal RSNA and 24 h urinary norepinephrine levels were significantly increased in T2D rats, suggesting that there was an increased overall sympathetic tone in the T2D rats (Table 1). However, there were no significant differences in basal MAP and HR between control and T2D rats.

### 3.2. Responses to Microinjection of Leptin into the ARCN or PVN

**Figure 2:**
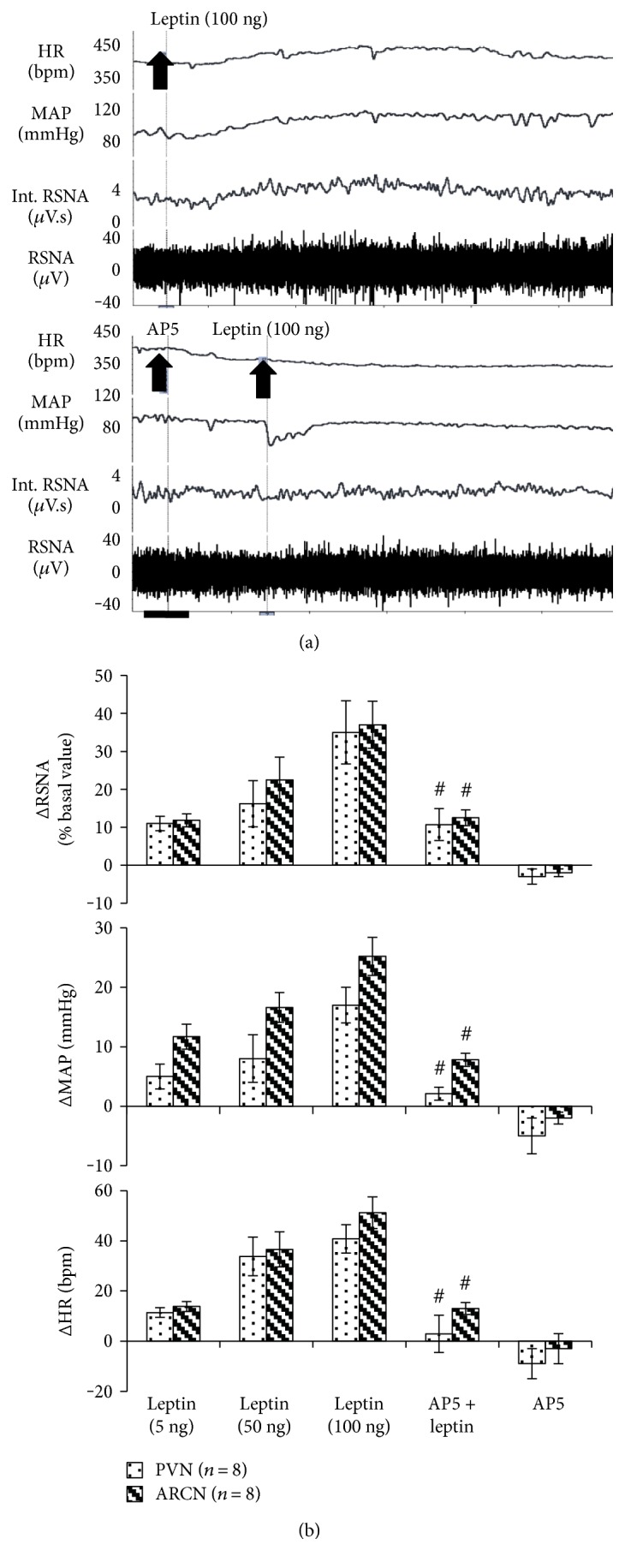
Renal sympathetic nerve activity (RSNA), mean arterial pressure (MAP), and heart rate (HR) responses to microinjection of leptin with/without preadministration of AP5 in the PVN or ARCN. ^#^*P* < 0.05 versus control group without AP5 administration. (a) A representative tracer of RSNA, MAP, and HR responses to microinjection of leptin with/without preadministration of AP5 in the PVN; bar = 1 min. (b) Mean changes in RSNA, MAP, and HR to microinjection of leptin (5–100 ng) with preadministration of AP5 (16 pmol) in the PVN or ARCN. ^#^*P* < 0.05 versus leptin group without AP5.

In anesthetized rats, microinjections of leptin (5 ng ~ 100 ng) into the ARCN or PVN induced increases in RSNA (ARCN: 37 ± 6%; PVN: 35 ± 8% at 100 ng), MAP (ARCN: 25 ± 3 mmHg; PVN: 17 ± 3 mmHg at 100 ng), and HR (ARCN: 51 ± 6 bpm; PVN: 41 ± 6 bpm at 100 ng) (Figure 2). Prior microinjections of NMDA receptor antagonist AP5 (16 pmol) into the ARCN or PVN significantly reduced a leptin-induced increase in RSNA (ARCN: 12 ± 2% versus 37 ± 6%, *P* < 0.05; PVN: 11 ± 4% versus 35 ± 8% at 100 ng, *P* < 0.05), MAP (ARCN: 8 ± 1 mmHg versus 25 ± 3 mmHg; PVN: 2 ± 1 mmHg versus 17 ± 3 mmHg at 100 ng, *P* < 0.05), and HR (ARCN: 13 ± 2 bpm versus 51 ± 6 bpm, *P* < 0.05; PVN: 3 ± 7 bpm versus 41 ± 6 bpm at 100 ng, *P* < 0.05).

### 3.3. Knockdown of the Leptin Receptor Inhibits NMDA-Induced Responses in the ARCN

**Figure 3:**
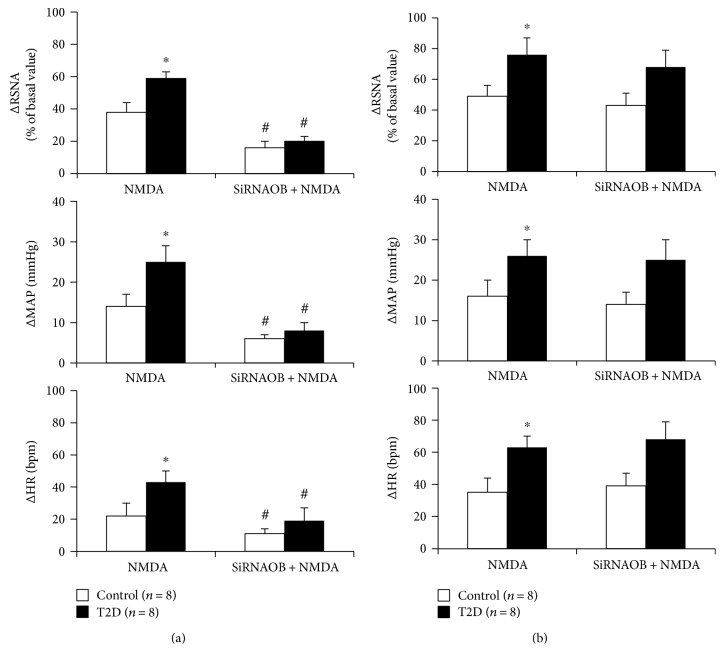
Renal sympathetic nerve activity (RSNA), mean arterial pressure (MAP), and heart rate (HR) responses to microinjection of NMDA (100 pmol) into the ARCN (a) or PVN (b) after knockdown of the leptin receptor with siRNA (siRNAOB) in the ARCN or PVN in control and T2D rats. ^∗^*P* < 0.05 versus control group; ^#^*P* < 0.05 versus group without siRNA knockdown.

Administration of NMDA (100 pmol) in the ARCN and PVN elicited increases in RSNA, MAP, and HR in both control and T2D groups (Figure 3). Microinjection of NMDA elicited significant increases in RSNA (ARCN: 38 ± 6%; PVN: 49 ± 7%), MAP (ARCN: 14 ± 6 mmHg; PVN: 16 ± 4 mmHg), and HR (ARCN: 22 ± 8 bpm; PVN: 35 ± 9 bpm) in control rats. The RSNA, MAP, and HR responses were significantly enhanced in T2D rats compared to the control rats, reaching 59 ± 4%, 25 ± 4 mmHg, and 43 ± 7 bpm, respectively, in the ARCN (Figure 3(a)) and 76 ± 11%, 25 ± 4 mmHg, and 63 ± 7 bpm, respectively, in the PVN (*P* < 0.05) (Figure 3(b)). Knockdown of the leptin receptor with siRNA in the ARCN and PVN significantly inhibited an NMDA-induced increase in RSNA in the ARCN (Figure 3(a)), but not in the PVN (Figure 3(b)), in both control and T2D rats (ARCN: 16 ± 4% versus 38 ± 6% in control and 20 ± 3% versus 59 ± 4% in T2D, *P* < 0.05), MAP (ARCN: 6 ± 1 mmHg versus 14 ± 3 mmHg in control and 8 ± 2 mmHg versus 25 ± 4 mmHg in T2D, *P* < 0.05), and HR (ARCN: 11 ± 3 bpm versus 22 ± 8 bpm in control and 19 ± 8 bpm versus 43 ± 7 bpm in T2D, *P* < 0.05).

### 3.4. Brain Histology

Figure 1() illustrates the brain histological data. Among the 32 injections within the ARCN or PVN area, 16 injection sites belong to normal group rats, 8 injection sites belong to control group rats, and 8 injection sites belong to T2D rats. A total of 6 injections missed intended injection sites.

### 3.5. Leptin Enhanced Intracellular Ca^2+^ Influx to Glutamate in Both Neuronal and Astrocytic Cell Lines

**Figure 4:**
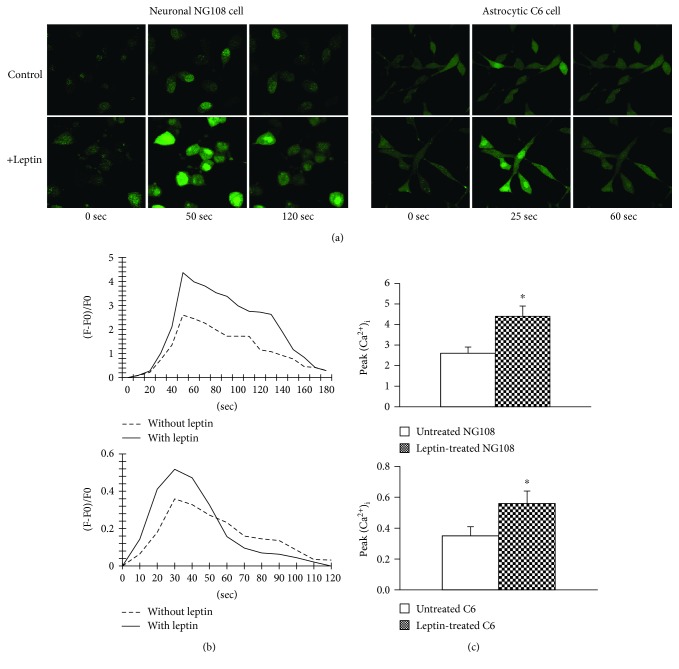
(a) Representative confocal images showing Ca^2+^ green fluorescent changes to glutamate (1 *μ*M) in neuronal NG108 and astrocytic C6 cells with/without leptin pretreatment. (b) Time-dependent responses of mean [Ca^2+^]_i_ to glutamate in each treatment group. (c) Summary data showing peak [Ca^2+^]_i_ in each treatment group. ^∗^*P* < 0.05 versus untreated group.

Figure 4 illustrates the effects of leptin on intracellular Ca^2+^ ([Ca^2+^]_i_) responses to glutamate in cultured neuronal NG108 and astrocytic C6 cells. Although basal [Ca^2+^]_i_ was not significantly altered by treatment with 5 *μ*M leptin, responses to glutamate were significantly affected. In the control group (untreated cells, *n* = 12), 1 *μ*M glutamate caused a rapid [Ca^2+^]_i_ increase that peaked at 2.6 ± 0.3 in neurons and 0.35 ± 0.06 in astrocytes, followed by a decline in [Ca^2+^]_i_ (Figure 4(b)). Compared with responses of untreated cells, the peak of the [Ca^2+^]_i_ response to glutamate was enhanced significantly in cells treated with leptin (*n* = 12). The magnitude of the peak response increased by 69% in leptin-treated neuronal cells (4.4 ± 0.5, *P* < 0.05) and 60% in astrocytic cells (0.56 ± 0.08, *P* < 0.05) (Figure 4(c)). The plateau phase of the response in both neuronal and astrocytic cells had no significant difference with leptin treatment.

### 3.6. Increased Leptin Receptor and NMDA NR_1_ Protein Expression in the ARCN and PVN in T2D Rats

**Figure 5:**
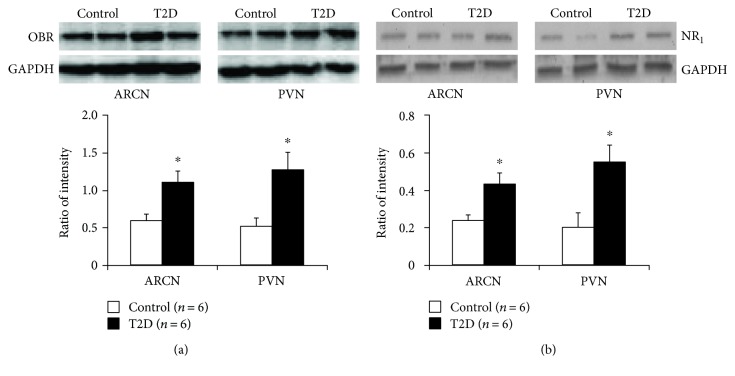
(a) Representative gel of leptin receptor (OBR) and mean protein expressions in the ARCN and PVN in control and T2D rats. ^∗^*P* < 0.05 versus control group. (b) Representative gel of NMDA receptor (NR_1_) and mean protein expressions in the ARCN and PVN in control and T2D rats. ^∗^*P* < 0.05 versus control group.

Western blotting analysis showed 100 kDa bands representing the leptin receptor and 115 kDa bands representing the NR_1_ receptor in the ARCN and PVN of control and T2D rats. T2D rats had significantly higher protein level of the leptin receptor (ratio of intensity: 1.06 ± 0.14 versus 0.54 ± 0.11, *P* < 0.05) and NR_1_ receptor (ratio of intensity: 0.43 ± 0.06 versus 0.24 ± 0.03, *P* < 0.05) in the ARCN (Figure 5). In the PVN, both leptin receptor and NR_1_ receptor protein expressions were also significantly increased in the T2D compared to the control rats (ratio of intensity—leptin receptor: 1.23 ± 0.21 versus 0.49 ± 0.11; NR_1_ receptor: 0.55 ± 0.09 versus 0.20 ± 0.08, *P* < 0.05).

**Figure 6:**
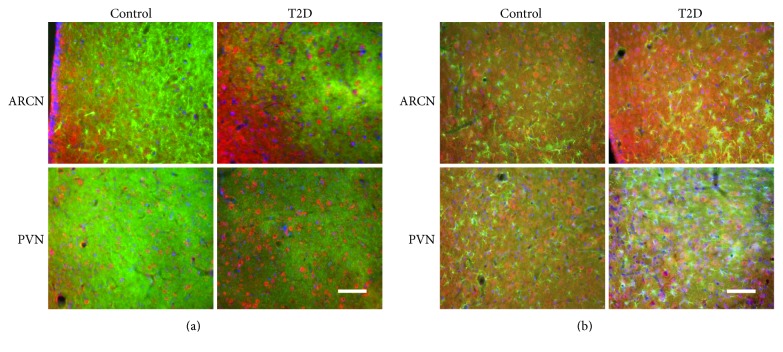
(a) Immunofluorescent photomicrographs from the sections of the ARCN and PVN region stained for the leptin receptor (red), neuronal marker microtubule-associated protein 2 (MAP2 in green), and 4′,6-diamidino-2-phenylindole (DAPI in blue) in a control and a T2D rat. (b) Immunofluorescent photomicrographs from the sections of the ARCN and PVN region stained for the leptin receptor (red), glial marker glial fibrillary acidic protein (GFAP in green), and DAPI (blue) in a control and a T2D rat. Bar = 100 *μ*m.

**Figure 7:**
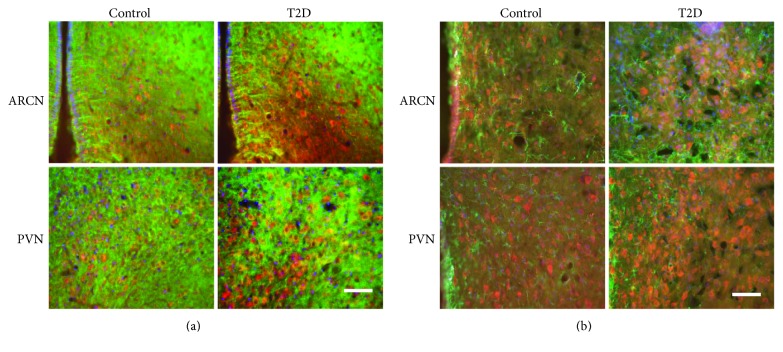
(a) Immunofluorescent photomicrographs from the sections of the ARCN and PVN region stained for NMDA receptor 1 (NR_1_ in red), MAP2 (green), and DAPI (blue) in a control and a T2D rat. (b) Immunofluorescent photomicrographs from the sections of the ARCN and PVN region stained for NR_1_ (red), GFAP (green), and DAPI (blue) in a control and a T2D rat. Bar = 100 *μ*m.

As an in situ confirmation of the alteration in the leptin receptor and NR_1_ receptor within the ARCN and PVN, the immunofluorescence for leptin receptors (Figure 6) and NR_1_ receptors (Figure 7) was found increased in the ARCN and PVN from rats with T2D compared with control rats. Both leptin receptor and NR_1_ receptor immunofluorescent signals were colocalized with the neuronal marker MAP2 within the ARCN and the PVN (Figures 6(a) and 7(a)). Leptin receptor immunofluorescent signals were also colocalized with the glial cell marker GFAP within the ARCN and the PVN (Figure 6(b)). However, NR_1_ receptor immunofluorescent signals were not colocalized with GFAP within the ARCN and the PVN (Figure 7(b)).

## 4. Discussion

In the present study, we have demonstrated that microinjections of leptin into the ARCN or PVN induce increases in RSNA, MAP, and HR. Prior microinjections of NMDA receptor antagonist AP5 blunted the leptin-induced increases in RSNA, MAP, and HR. Knockdown of leptin receptor expression with siRNA inhibited NMDA-induced increases in RSNA, MAP, and HR in the ARCN but not in the PVN. In *in vitro* studies, preincubation of neuronal NG108 cells with leptin induced a robust increase in intracellular Ca^2+^ green fluorescence when the cells were challenged with glutamate. Furthermore, in high-fat diet and low-dose STZ-induced T2D rats, we found that leptin receptor and NMDA NR_1_ receptor expressions in the ARCN and PVN were significantly increased. Taken together, these results show that within these hypothalamic nuclei, leptin-glutamate signaling regulates the sympathetic activation. This may contribute to the sympathoexcitation commonly observed in obesity-related T2D.

Adipose tissue-released leptin exerts an influence on many physiologic processes, including food intake, thermoregulation, fertility, sympathetic nerve activation, renal function, blood vessel tone, and blood pressure. The role of leptin in activating sympathetic drive has been highlighted in many reviews [23–25]. In the whole animal studies, we have observed that microinjections of leptin into the ARCN or PVN induce an increase in RSNA, MAP, and HR. This is consistent with reports that intracerebroventricular- (ICV-) administered leptin increases sympathetic nerve activity to the kidney [26, 27].

The hypothalamic ARCN has been shown to regulate energy balance and blood pressure [27]. Stimulation of the ARCN with leptin and glutamate elicits increases in RSNA and MAP [13]. The hypothalamic PVN is one of the major preautonomic centers that directly control sympathetic outflow in the central nervous system [28]. Stimulation of PVN has been shown to elicit an increased discharge from several sympathetic nerves, including renal, adrenal, and splanchnic nerves [29–31]. Studies indicate that the ARCN is a gateway for the action of insulin and leptin on sympathetic activity [32]. The ARCN-to-PVN circuit involved in energy homeostasis is directly and tonically controlled by leptin in a multinodal fashion [5]. Neurons in the ARCN express leptin receptors and play an important role in transmitting the leptin signal to the PVN neurons. The ARCN-PVN projection appears to be overactive in diabetic rats [33].

In the central nervous system, leptin appears to exert its effects on sympathetic activity and blood pressure through a number of mediators. A novel interaction between angiotensin-II and leptin in the control of sympathetic nerve activity, brown adipose tissue thermogenesis, and body weight has been reported recently [34, 35]. Leptin also regulates PVN neurons indirectly, by binding to receptors in the ARCN and regulating the release of neural effectors like glutamate from first-order neurons onto preautonomic neurons in the PVN [16]. Glutamate NR_1_ is markedly augmented in the PVN of diabetic rats [15]. The precise mechanisms of leptin-glutamate signaling within the hypothalamus that lead to sympathoexcitation in T2D remain to be determined.

Both leptin and glutamate are important neuromodulators in the central nervous system. High glutamatergic tone has been shown in several hypersympathetic disease conditions, such as diabetes, hypertension, and chronic heart failure [14, 15, 36]. Our data has shown that central leptin-induced increases in RSNA, MAP, and HR were attenuated by glutamate NMDA receptor antagonist AP5 in both the ARCN and PVN. These data support the concept of a brain leptin-glutamate interaction in which the brain glutamate mechanism facilitates leptin-induced increases in sympathetic nerve activity. Both the ARCN and PVN are possible sites for the leptin-glutamate interaction. When we knock down the leptin receptor, a central NMDA-induced increase in RSNA, MAP, and HR was attenuated in the ARCN but not in the PVN. This suggests that leptin facilitates NMDA-induced increases in sympathetic nerve activity in the ARCN. However, in the PVN, NMDA-mediated sympathetic activation is more dominant and appears to be likely more independent of leptin signaling.

By confocal calcium image, in cultured neuronal NG108 cells and astrocytic C6 cells, we have observed that preincubation with leptin for 24 hours induced a robust increase in intracellular Ca^2+^ green fluorescence when the cells were challenged with glutamate compared to the control group without leptin pretreatment. The responses occurred immediately after leptin administration followed by a rapid return to baseline. Leptin caused a robust increase in glutamate-induced calcium signaling in both cultured neurons and astrocytes, confirming functional changes in neurons and astrocytes induced by leptin via a glutamate receptor.

The hypothalamus is an extremely heterogeneous tissue comprised of astrocytes, oligodendrocytes, microglia, endothelial cells, ependymal cells and numerous neuronal subgroups [37]. Astrocytes have recently emerged as an active component of various complex central mechanisms, and it is now clear that their role in the brain is by no means limited to just providing structural and metabolic support to neurons. Astrocytes can affect neuronal activity in a variety of ways. This may include the release of glutamate, ATP, or other signaling molecules [38]. Astrocytes are possible cellular substrates of angiotensin (1–7) that affect local metabolism and microcirculation in the RVLM, resulting in changes in the activity of RVLM presympathetic neurons and hence blood pressure [39]. Regulation of tonic GABA inhibitory function, presympathetic neuronal activity, and sympathetic outflow from the PVN is shown to be modulated by astrocytic GABA transporters [40]. Leptin receptor mRNA and protein are observed in both astrocytes and neurons in the rat hypothalamus [41]. It has been reported that metabolic changes in obese mice can rapidly alter leptin receptor expression and astrocytic activity. The leptin receptor is responsible for leptin-induced calcium signaling in astrocytes [42].

In the T2D rat model, we have observed that 12–14 weeks of HFD and single low-dose STZ injection produces hyperglycemia, hyperleptinemia, hyperlipidemia, and insulin resistance in the rat. Hyperleptinemic condition might be implicated in generating the elevation of sympathoexcitation in T2D rats. We have observed that leptin receptor and NMDA NR_1_ protein levels in the ARCN and PVN tissues were upregulated in T2D rats compared to the control rats. This result suggests that upregulation of leptin receptors and NMDA receptor within the hypothalamus may be one possible mechanism for the enhanced endogenous leptin-glutamate-mediated excitatory action on sympathetic outflow in T2D. Furthermore, double-labeling immunohistochemistry analysis showed that both neurons and astrocytes in the hypothalamus expressed leptin receptors while NMDA NR_1_ receptors were only localized on the neurons. The results imply alterations of leptin receptor expression and astrocytic activity within the ARCN and PVN in T2D rats. Determining exactly which specific cell types activate leptin signaling may yield novel and critical clues to the mechanisms related to the altered neurohumoral drive during T2D.

## 5. Conclusion

These studies provide evidence that within the hypothalamic nuclei, leptin-glutamate signaling regulates sympathetic activation. This altered mechanism/s within the PVN may contribute to the increased renal sympathetic neural activity observed in T2D. These results provide a potential target for the treatment of enhanced sympathoactivation commonly observed in T2D.

## Figures and Tables

**Table 1:** General characteristics of control and T2D rats.

	Control (*n* = 16)	T2D (*n* = 16)		Control (*n* = 16)	T2D (*n* = 16)
Body weight (g)	427 ± 24	485 ± 29^∗^	Plasma glucose (mmol/L)	5.3 ± 0.3	17.2 ± 1.5^∗^
Retroperitoneal fat pad (g)	5.1 ± 0.3	8.7 ± 1.6^∗^	Plasma insulin (mU/L)	13.7 ± 1.3	13.9 ± 1.1
Epydidimal fat pad (g)	8.2 ± 1.2	9.5 ± 1.4	Insulin sensitivity index	−4.28 ± 0.14	−5.47 ± 0.28^∗^
Brown adipose tissue (g)	1.2 ± 0.2	0.3 ± 0.1^∗^	Plasma leptin (ng/mL)	304 ± 26	479 ± 39^∗^
Basal MAP (mmHg)	90 ± 4	93 ± 5	Basal heart rate (beat/min)	350 ± 16	362 ± 20
Basal int. RSNA (*μ*V·s)	3.2 ± 0.3	4.7 ± 0.4^∗^	24 hrs urine NE (*μ*g)	183 ± 36	403 ± 50^∗^

Values are mean ± SE. ^∗^*P* < 0.05 versus control group. MAP: mean arterial pressure; int. RSNA: integrated renal sympathetic activity; NE: norepinephrine.
